# Robust Policy in Times of Pandemic

**DOI:** 10.1007/s10272-021-0961-1

**Published:** 2021-04-06

**Authors:** Jan Willem Van den End, Yakov Ben-Haim

**Affiliations:** 1 Department of Finance, ECO/FIN, Vrije Universiteit Amsterdam, De Boelelaan 1105, 1081 Amsterdam, HV, Netherlands; 2 Faculty of Mechanical Engineering, Technion - Israel Institute of Technology, Dan and Betty Kahn Building, 32000 Haifa, Israel

## Abstract

The pandemic exposes policymakers to fundamental uncertainties about future economic scenarios. While policymakers have to act forcefully to mitigate the impact on the economy, these conditions call for policy strategies that are also robust to uncertainty. This article compares two concepts of robust strategies: robust control and robust satisficing. It argues that a robust satisficing strategy is preferred and shows that the crisis responses of governments and central banks in Europe share features of robust satisficing in several dimensions.

## The pandemic creates fundamental uncertainty

The pandemic is an unprecedented shock with a sizeable downward skew. The event is not reflected in past data, so its social and economic impact cannot be quantified based on known probability distributions. This makes any forecasts fundamentally uncertain, as explained long ago by Knight (1921).

The economic impact of the pandemic is fundamentally uncertain for several reasons. We do not know how many waves of COVID-19 will affect our societies or for what duration immunity is obtained after vaccination. The impact of the virus on social and economic developments has the potential to change the economy's productive capacity either permanently or for a sustained period. Such shocks to the economy are associated with radical, or Knightian, uncertainty (Issing, 2002). In these circumstances economic policymakers need strategies that are robust to fundamental uncertainty.

**Jan Willem Van den End**, De Nederlandsche Bank, Amsterdam; and Vrije Universiteit Amsterdam, Netherlands.

**Yakov Ben-Haim**, Technion – Israel Institute of Technology, Haifa, Israel.

## Robust strategies

Two distinct concepts of robust strategies have emerged: robust control and robust satisficing. A robust control approach (Hansen and Sargent, 2008) assumes that an aggressive strategy in times of fundamental uncertainty helps minimise the likelihood and impact of potential worst-case outcomes. For policy responses to the coronavirus crisis, this could imply a massive monetary and fiscal response to prevent a particular worst-case economic scenario from happening.

Robust control strategies in economics are derived from control theory in engineering and the min-max concept (Wald, 1945): minimising the impact of a maximally adverse situation. Although the goal of avoiding the worst outcome seems attractive, in economics it is difficult to realistically identify meaningful worst cases. While engineers can sometimes usefully identify worst cases, economic scenarios are far more complex and multi-facetted, making worst cases hard to formulate reliably.

The difficulty of identifying realistic worst cases in times of fundamental uncertainty and the need to achieve specific policy objectives motivate the robust satisficing strategy. The central concept is satisficing, which means achieving acceptable outcomes or, equivalently, meeting critical goals. As pointed out by Simon (1956, 1983), this is distinct from outcome-optimising that aims to achieve the best possible outcome. It is also different from min-max that seeks to ameliorate the worst-case outcome. The satisficing approach recognises that fundamental uncertainty precludes both outcome-optimisation and worst-case minimisation. It identifies critical goals that must be achieved and tries to achieve them as reliably as possible. The resulting policy response can either be moderate or aggressive, depending on the critical goals.

## Policy trade-offs

Economic policymakers nowadays face a range of challenges: output losses, increasing unemployment, looming bankruptcies, rising budget deficits and debts, and so on. These challenges are preferably addressed together and this may involve trade-offs between them. For instance, supporting economic recovery may exacerbate budget deficits, and support to firms may weaken long-term productivity. But the overarching trade-off is between the economic goals and the management of the deep underlying uncertainty.

As the goals become more demanding, like aiming at a V-shaped recovery from a lockdown, the vulnerability to adverse surprise increases as well. This is because greater aspirations can fail in more ways than modest aspirations. The proactive policymaker must identify the essential goals and achieve them as reliably as possible, despite the potential for severe adverse surprise. This may call for a precautionary approach, though not aimed at minimising the likelihood of a particular worst-case scenario.

Critical goals can usually be satisfactorily achieved in diverse ways precisely because one is not aiming at a unique optimal outcome. Various alternative policies would satisfice (but not necessarily optimise) the outcome. This plurality of satisficing policies creates an additional degree of freedom in policy formulation: something other than the outcome can be optimised, while the outcome itself is satisficed. Specifically, the policymaker can optimise the robustness to uncertainty and surprise, while satisficing the quality of the outcome. The robust satisficing approach balances the ambitiousness of the goals and the robustness to surprise.

A common misconception is that robust satisficing is inherently cautious or slow to act. This results from misinterpreting the concept of satisficing, which means “meeting *critical* goals”, as if it meant “meeting *minimal* goals”. The critical goals may be very ambitious, and probably should be very ambitious in extreme circumstances. This implies that robust satisficing could also advise vigorous policies to support the economy in the current crisis.

Consider the depression of the 1930s: economists agree that US fiscal and monetary policymakers did too little, too late (Fishback, 2010). Policymakers at the time could have adopted the ambitious satisficing goal of reducing unemployment from 25% to 15%, and then to 5%, over two or three years, for instance. Satisficing goals like these are not optimisations, such as “minimise unemployment” or “minimise the duration of excess unemployment”. But they would have been highly ambitious. Once satisficing goals are defined, the policymaker adopts the most robust policy for achieving them.

## Proxies for robustness

Both robust control and robust satisficing strategies can be evaluated in terms of their strength in several proxies for robustness. The robustness of a policy strategy is the ability to achieve specified goals, despite the occurrence of unanticipated adverse events, like another surge of COVID-19 infections. Robustness is a multifaceted attribute of a policy (Ben-Haim, 2010, 2018; Ben-Haim and Demertzis, 2016). We identify six conceptual proxies for robustness to uncertainty. They overlap to varying degrees, but each reflects a different aspect of robustness.

*Resilience*: rapid recovery of critical functions. Some adverse events interrupt critical economic functions. A policy that enables rapid restoration of such functions has resilience to uncertainty.

*Redundancy*: multiple alternative solutions. A policy has redundancy if alternative tools can be brought to bear in responding to surprise.

*Flexibility*: rapid modification of tools and methods. A policy is flexible if it can be altered and new effects achieved at short notice.

*Adaptiveness*: modification of tools and methods over a longer time. Flexibility is a short-term attribute, while adaptiveness of a policy refers to its long-term ability to adjust to changing circumstances.

*Margin of safety*: excess of benefits. A policy has a margin of safety if it provides policy space to act in unanticipatedly stressful circumstances.

*Comprehensiveness*: interdisciplinary system-wide coherence. A policy is comprehensive to the extent that it addresses diverse facets of the challenge. A comprehensive policy assesses the impact in different sectors, over different horizons, while also taking into account the actions of other players.

## Assessment of policy strategies

The proxies for robustness can be used to evaluate both robust control and robust satisficing. A strategy is preferred if it is strong in most or all of the proxies and hence robust for achieving specified critical goals. While a robust control strategy likely scores high on at least some of the six criteria, this strategy also has downsides in a state of fundamental uncertainty.

First, it can give a false sense of being in control, while the future dynamics of the economy are unknown. It does not seem sensible to design policies for selected worst cases about which one knows very little (Sims, 2001). This may reflect policymakers' overconfidence in achieving the objective.

Second, pre-committing to particular future actions will reduce adaptiveness and the margin of safety. It can also constrain the possibility of other interventions. This may undermine the policymaker's credibility.

Third, suggesting that policies are in place to minimise the impact of a worst-case outcome may reduce resilience. It can incentivise market participants to change their behaviour, causing the economy to deviate from desired outcomes.

To some extent these pitfalls may also accompany robust satisficing strategies. A robust satisficing strategy may create a false sense of control if it entails a long-term commitment, although this would be in the context of flexibility and adaptiveness. A robust satisficing policy is preferable to min-max robust control in several aspects.

- It is *flexible* and has *redundancy* in terms of alternative measures by aiming at the objective in diverse ways, precisely because there is not just one optimal outcome.
- It scores high on *adaptiveness*, since it works under flexible assumptions by acknowledging the prominence of uncertainties of scenarios and outcomes.
- It scores high on *resilience* because it tries to achieve the objective as reliably as possible, thereby implicitly nurturing critical functions of the economy.
- By not pre-committing specific resources to cope with specific worst-case scenarios, it preserves a *margin of safety* (also in terms of reputation and credibility) to respond to future surprises.
- It scores high on *comprehensiveness* because a satisficing strategy has a modest view on what the policymaker can achieve and acknowledges that actions by other economic agents are also needed.

Robust control and robust satisficing can also be compared on a more conceptual level with the four aspects of decision making shown in Figure 1. The figure displays the methodological preference for robust satisficing.

Figure 1
**Conceptual comparison**

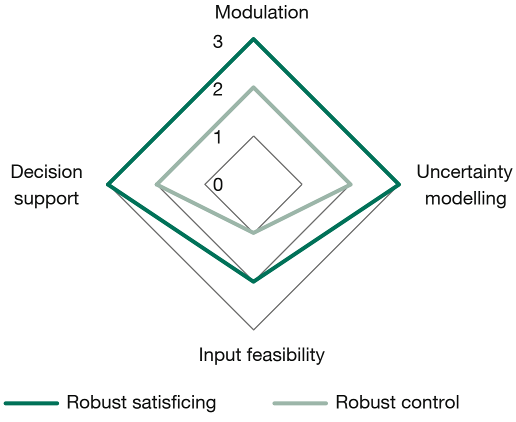

Source: Authors' own elaboration.

*Modulation*: variation in the policy response. Robust control is inherently aggressive because it is designed to respond to postulated worst cases. Robust satisficing on the other hand allows the policymaker to explore the full range of uncertainty by examining the trade-off between robustness to uncertainty and quality of outcome. This implies that robust control has medium modulation, while robust satisficing has large modulation.

*Uncertainty modelling*: dexterity in modelling and managing uncertainty. Robust control is a relatively blunt strategy: the wide and rich range of contingencies and scenarios is embedded in postulated worst cases. Robust satisficing directly addresses uncertainty in its myriad dimensions. Hence, robust control is medium and robust satisficing is high in uncertainty modelling.

*Input feasibility*: ease of providing required input data. Robust control requires estimated worst cases, about which one knows little and which are extremely hard to calibrate. Robust satisficing requires that the policymaker specifies essential or critical outcomes. The reliability and feasibility of the robust control inputs are low, while those of robust satisficing are medium, limited primarily by the knowledge of the policymaker's priorities.

*Decision support*: providing conceptual tools to support deliberation and selection of policy. Robust control tends to be a one-way street, portraying the ability of policy alternatives to manage the postulated worse cases. Robust satisficing encourages the policymaker to have alternative policy options available. Hence, robust satisficing is strong in decision support, while robust control is moderate.

## Evaluation of the European policy response

Economic policy measures taken to address the economic fallout of the COVID-19 crisis can be assessed by their robustness to uncertainty. Governments in Europe have provided unprecedented financial support to households and firms to compensate for their reduced income and turnover in the lockdowns. The measures range from direct income support to guarantees on bank loans that indirectly support the liquidity position of firms (see IMF, 2020, for an overview). Extended liquidity supply and asset purchases by the European Central Bank (ECB) have supported banks and financial markets in providing the necessary financial services to the economy.

In terms of the robust satisficing concept, governments and central banks have aimed at critical goals that must be achieved in a fundamentally uncertain market environment. Governments aim at keeping businesses afloat that are viable in the long run. This also supports jobs and prevents long-term economic damage due to insolvencies and hysteresis effects. Such effects can be related to long-term unemployment and loss of company-specific knowledge. The goal has been to keep these losses at tolerable – though not explicitly minimal – levels. Central banks and supervisors have supported the critical functions of the financial system, while being vigilant for financial amplification effects that may occur through adverse spillovers from the corporate sector to financial institutions.

The policy measures meet several proxies for robustness and aspects of decision making discussed above. The measures contribute to *resilience* by preserving critical functions of the economic and financial system to prevent long-term economic damage. They also meet the *redundancy* criterion, since government and central banks have used alternative tools to support the economy. From the onset of the pandemic, it was unclear which measures were most effective. This motivated authorities to roll out support packages that contained a host of measures and support schemes. Such packages provided the necessary *flexibility* to modify the responses and tools to changing circumstances. This is in line with the *modulation* principle in decision making, which advocates variation in the policy responses.

In most countries the government support measures are also *adaptive* over time, which is the case if their use and conditions move in sync with the economic situation. It is important that the process of winding down the support measures in a later stage remains cognisant of the persisting uncertainties in the economic and medical spheres. Adaptability is also part of those government support schemes that provide incentives to adjust to the new business realities. Keeping in place the support measures too long may be costly if the economic structure changes due to the COVID-19 crisis. It is not cost-efficient if firms and sectors that will have a smaller market share in the future continue to be supported. The private sector itself also shows adaptability owing to learning effects from the first lockdowns in the spring of 2020. Firms have learned how to adjust in a lockdown, which has limited the economic damage in subsequent lockdowns (DNB, 2020).

The measures by governments and central banks have been unprecedented in terms of size and scope. This has provided a *margin of safety*. The increase of fiscal deficits and public debts are a reflection of the policy space that has been created to act in these unanticipated stressful circumstances. In the euro area countries, fiscal spending in the form of transfers and subsidies to firms and households amounts to about 4.5% of GDP (ECB, 2020). As a consequence, the average euro area debt ratio is projected to peak in 2021 at almost 100% of GDP. It reflects the role of the public sector as insurer against extreme tail risks such as the pandemic. It underlines the importance of sufficient policy space, created by building up financial buffers in normal circumstances. The three safety nets endorsed by the European Council for workers, businesses and sovereigns also provide important funding support. The fiscal response is supported by the accommodative monetary policy of the ECB. Since March 2020, the ECB has purchased government bonds and corporate bonds through the Pandemic Emergency Purchase Programme to preserve favourable financing conditions until it is judged that the coronavirus crisis phase is over. The current fiscal and monetary policy mix displays interdisciplinary system-wide coherence, as the fiscal and monetary measures work in the same direction. This meets the *comprehensiveness* criterion of robustness.

The ECB's monetary policy measures are based on two alternative scenarios for the economy, in addition to the baseline scenario (ECB, 2020). The scenarios vary according to different assumptions about the pandemic and about the economic response. In that sense, the scenarios are a tool for uncertainty modelling and are supportive to managing uncertainty. The scenarios are adjusted in the course of time to include new incoming information. This is in line with the input feasibility aspect of a decision making process that is robust to fundamental uncertainty. The scenarios provide conceptual tools to support the deliberation and selection of monetary policy. As a decision support instrument they stimulate the policymaker to consider alternative policy options available. If the rollout of vaccinations leads to sufficient herd immunity by the end of 2021, the economy will start to function under more normal circumstances (Lagarde, 2020). That could mark the end of the emergency phase and of the large uncertainties under which policymakers have to make their decisions.

## Conclusion

The pandemic has created fundamental uncertainties about the economic outlook. It has challenged policymakers to define a strategy that is robust to future unexpected dynamics in social, medical and economic spheres. We conclude that a robust satisficing strategy that maximises robustness to uncertainty and satisfices policy goals is preferred over a robust control strategy that minimises the impact of postulated worst cases. This conclusion is motivated by the huge uncertainties related to the COVID-19 crisis that makes the identification of realistic worst cases highly unreliable.

The policy strategies of governments and central banks in Europe share the distinguishing features of robust satisficing in several dimensions. The unprecedented policy responses have created a margin of safety and display an interdisciplinary and system-wide coherence that is needed to effectively address the economic challenges of the coronavirus crisis. It supports the achievement of critical goals, such as preserving jobs and activities in viable businesses in circumstances characterised by fundamental uncertainty.
